# Cabin as a Home: A Novel Comfort Optimization Framework for IoT Equipped Smart Environments and Applications on Cruise Ships

**DOI:** 10.3390/s19051060

**Published:** 2019-03-02

**Authors:** Massimiliano Nolich, Daniele Spoladore, Sara Carciotti, Raol Buqi, Marco Sacco

**Affiliations:** 1 Department of Engineering and Architecture, University of Trieste, Via Alfonso Valerio 6/3, 34127 Trieste, Italy; scarciotti@units.it (S.C.); rbuqi@units.it (R.B.); 2 Institute of Intelligent Industrial Technologies and Systems for Advanced Manufacturing, National Research Council, Via Previati 1/E, 23900 Lecco, Italy; daniele.spoladore@stiima.cnr.it (D.S.); marco.sacco@stiima.cnr.it (M.S.)

**Keywords:** IoT, holistic comfort, decision-making, comfort optimization, cruise cabin, cabin comfort

## Abstract

The international tourism competition poses new challenges to the cruise sector, such as the achievement of the tourists’ satisfaction and the increase in on board comfort. Moreover, the growing sophistication of tourists’ needs leads to a more user-centric touristic offer. Consequently, a personalized cabin environment, which fits the users’ activities and their characteristics, could be a plus value during the cruise vacation. These topics, however, are strictly connected with the diffusion of digital technologies and dynamics, which represent the tools to achieve the goal of a customized on-cruise experience. This paper presents E-Cabin, a novel Internet of Things (IoT) framework architecture that has at its core a reasoning system tuned on data gathered from the environment and from each specific passenger and the activities he/she performs. The framework leverages on knowledge representation with ontologies and consists of a publisher–subscriber communication framework that allows all of the IoT applications to use the reasoner and the provided ontologies. The paper demonstrates the proposed system in a demo cruise cabin where, by using the E-Cabin application, it is possible to set various atmospheres based on the users and activities occurring in the cabin.

## 1. Introduction

The tourism industry is becoming more and more competitive, as tourists can choose their stay by using a variety of web-based portals. In particular, the cruise sector is growing faster than other industries in tourism [1] and is a very competitive branch of this industry. The focus of this competition regards the exploitation of the most novel technological advances in cruise ship building [2]. The competition also encompasses the quality of services a cruise cabin can deliver to its guests; in this regard, several attempts to improve comfort have been investigated. The reduction in noise and vibration is a research area that gathers a lot of attention, especially in naval engineering [3,4]. Another relevant aspect often investigated for cruise cabin regards the pricing policy and the advertising strategies adopted [5,6,7].

However, indoor comfort for cabins has been very little researched. Kwortnik [8] analyzed the “shipscape”, defined as the leisure cruise service environment. Collecting data from 260 cruise customers, the author revealed how the cruise experience—from a passenger’s point of view—is something between the theme park and the cruise. Using 100 questionnaires, Goujard [9] assessed acoustic comfort in relation to other factors (temperature and light); acoustics was found to be a significant criterion of global comfort experience and noises (squeaking, cracking, clattering, etc.) were classified as disturbing for the passengers. In 2017, Jinjin [10] investigated the role of color design in cabin’s interiors, concluding that simple and bright colors can be of some influence on the passengers’ mood. Recently, a survey regarding procedures and notations for passengers’ comfort quantification has been conducted [11], highlighting how vibrations, noise, indoor temperature and lighting are relevant factors in determining comfort, although they are addressed in different ways in classifications. Moreover, a preliminary description of cruise cabin comfort has been proposed in Ref. [12], defining a cruise cabin comfort ontology, and the basic definition of how to use a reasoner for cabin comfort is described in Ref. [13]. Nevertheless, indoor comfort is a widely debated topic in several disciplines that emerged in the last several decades, such as Ambient Assisted Living (AAL) [14], Ambient Intelligence (AmI) [15] and Context Awareness (CA) [16]. These disciplines consider the use of interconnected sensing technologies, knowledge management, data acquisition from the environment and the inhabitants occupying specific living environments. The aim of the technologies involved by these disciplines is to improve the comfort of inhabitants by providing the possibility to overcome some person-related limitations through technology and to make the living environments “smarter”, i.e., to use Internet of Things (IoT) technologies to foster automatic actuation of environmental components (such as opening windows, turning on/off the air conditioning system fostering energy saving, etc.) [17,18,19]. Therefore, these disciplines address several domains of knowledge—healthcare, comfort, sensors, artificial intelligence, mobile computing, etc.—and have been extensively investigated in research, with particular attention to the IoT framework [20,21]. In fact, there is a growing interest in IoT equipped smart environments, as the technological offer is growing and the costs of the implementations are decreasing. This work introduces the E-Cabin reasoning framework, one of the outputs of a project dedicated to the development of a smart cruise cabin, in which AmI and CA technologies work in a synergistic way inside a IoT framework to provide passengers with customized indoor comfort and to foster energy saving behaviours.

The main contributions of this paper are related to the definition of a modular decision-making system (DMS) based on predefined semantic classifications together with the usage of such system on a cruise cabin, as a set of comfort tests has been carried out on scenarios and use cases defined on a real cruise cabin placed in our laboratory (located inside the University of Trieste, Italy). The cruise cabin environment has been provided by Fincantieri S.p.A. (Trieste, Italy), a company that builds cruise ships for different shipholders. Moreover, all the scenarios and the use cases presented in this work have been defined with the collaboration of Fincantieri S.p.A.

The remainder of this work is organized as follows: Section 2 presents some of the most relevant works related to the field of IoT technologies and knowledge engineering adopted to make a living environment “smarter”. Section 3 delves into the E-Cabin decision-making framework, describing its architecture, the technologies involved (with particular focus on the reasoning-enabling framework) and its general structure. Section 4 introduces some use cases to depict E-Cabin’s possibilities and functioning in plausible contexts, encompassing passengers’ specific needs, the activities they want to perform and energy saving procedures. Section 5 describes the E-Cabin application, the mobile-based Graphic User Interface (GUI) that allows the passengers to use E-Cabin’s features. Section 6 illustrates a subjective study whose aim is to assess the perceived utility of E-Cabin—i.e., whether the passengers find beneficial to have a cabin able to change and adjust the indoor comfort taking into account their activities and characteristics.

## 2. Related Work

Considering that comfort has been acquiring a growing importance due to the spreading of AAL, AmI and CA technologies, this section investigates the most relevant works related to (a) ontology developing for indoor comfort; (b) ontology for the description of the person and his/her status in the context of a smart environment; and (c) AmI, AAL and CA systems leveraging ontologies as enabling technology to provide indoor comfort customization and activity personalization.

The issue related to modelling indoor comfort metrics has been widely addressed in literature; however, any ontology emerged as a “standard” model, and neither was the most adopted. Due to the variety of the comfort metrics and the different research needs, each research group relied on ontologies developed for specific purposes. Flexergy [22], a model developed to describe the sustainable comfort, is focused on the representation of sensors, actuators and device, connecting the possibility to provide personalized comfort to energy saving practices. Frešer et al. [23] modelled three indoor comfort metrics (CO${}_{2}$ concentration, humidity rate and temperature) in an Ontology Web Language (OWL) [24] model with Semantic Web Rule Language (SWRL) [25] rules with the aim of triggering actuations. Based on the ISO 7730:2005, Adeleke et al. [26] formalized an Indoor Environmental Quality semantic model in order to monitor indoor air quality to identify potential risks for inhabitants’ health. Some comfort metrics are also described in ThinkHome [27], a smart home system that leverages on ontological representations of knowledge to infer the most suitable comfort parameters with regard to inhabitants’ age and gender. In the Smart Home Simulator [28], the authors exploited ontologies to represent comfort metrics (CO${}_{2}$ concentration, humidity rate, indoor temperature, illuminance) in order to evaluate whether these are comfortable or uncomfortable for the dwellers.

Since smart environments—especially smart homes—are expected to provide tailored services to their end-users, the modelling of the person acquires a pivotal importance. The description of human activities within a smart environment has been investigated by Ni et al. [29], where ontologies are used to represent the residents in their living contexts. Semantic-based technologies have also been exploited to describe dwellers’ health conditions, in order to foster actuation of services according to the specific needs of smart environments’ residents. RoomFort [30]—an ontology based system for indoor comfort personalization for hotel rooms—leverages both on ontologies describing the health condition of a business traveller (using the International Classification of Functioning, Disability and Health (ICF) [31]), comfort metrics and a list of activities a business traveler can perform in the hotel room. The ICF-based approach for a health condition description is starting to emerge in several works, which combine semantic-based technologies to foster AmI and CA. In Ref. [32], the health condition description is conducted with ICF to provide a customization of the smart home services; in Ref. [33], the ICF-based health condition is the cornerstone for the reconfiguration of living environments in an AAL context.

Apart from the above-mentioned Smart Home Simulator and RoomFort, several others’ recent AmI, AAL and CA systems leveraging on ontologies can be traced in literature. Sezer et al. [34] described a simulated sensors network exploiting a smart home ontology, which acts as a collector of data gathered by the sensors. Meng et al. [35] presented a rule-based service customization strategy aimed at enhancing home environments; in this work, ontologies are used to define rules for customization of services. In the field of CA for clinical support, Andreadis et al. [36] presented Dem@Home, a CA monitoring system for dementia caring at home developed to improve independent living; ontologies here are extensively employed to represent sensor observations, activity recognition and problem detection. Tila et al. [37] used semantic modelling to provide semantic interoperability among different data and to back-up the deployment of an IoT system for indoor environment control. Finally, a multi-level smart city architecture—leveraging both semantic-based technologies and wireless sensors—is presented in Ref. [38], where OWL ontologies are adopted to enrich raw data acquired by the sensors.

## 3. The Proposed Novel Decision Making Framework for Smart Environments

The E-Cabin framework leverages ontological representations of some domains of knowledge to enable reasoning for indoor comfort customization and for environmental actuation triggering. The proposed reasoning system can turn an IoT equipped environment into an autonomous and automatic system capable of automatic adaptation of the environment, also taking into account the passengers’ personalized concept of comfort in a holistic way. Due to its aims, E-Cabin’s relevant domains of knowledge are not limited to sensors, but encompass the passenger—with his/her health condition, preferences, activities, etc.—and the cabin environment.

In this paper, we present a DMS deriving from the combination of semantic reasoning over a set of domain ontologies [39]. The ontologies constitute the framework’s knowledge base and provide a formal description of relevant pieces of knowledge for E-Cabin; the result of this combination consists in the capability of improving passengers’ perceived comfort inside their cruise cabin. The proposed reasoning system is based on the following elements:

**A.**  ontology related to the IoT devices in the smart environment;
**B.**  ontology related to the person;
**C.**  ontology related to comfort.

As stated in Section 2, in literature, there are several ontologies that can represent smart environments equipped with IoT devices. Such descriptions are commonly adopted to describe the working, monitoring and acting phases of the devices, and the monitoring and optimization of power consumption are a classical issue that is addressed. For instance, W3C-endorsed Semantic Sensor Network (SSN) ontology [40] was developed in 2012 to describe sensors and their observation. Following the NeOn Methdology [41] for ontological resources reuse and development, E-Cabin framework reuses part of the SSN ontology for the description of IoT devices deployed in the smart environment, while adopting the ontology design patterns provided in Ref. [30] for the modelling of the passenger and his/her health condition. Finally, for indoor comfort metrics, E-Cabin monitors air quality (i.e., CO${}_{2}$ concentration), illuminance and indoor temperature and humidity leveraging on a domain ontology developed from scratch.

This last ontology acts as a link between the environment and the person and contains comfort metrics evaluated according to the legislation together with holistic parameters that can help the automatic adaptation of the perceived comfort. The ontologies involved in E-Cabin are described in the following subsections (from Section 3.2, Section 3.3 and Section 3.4).

The proposed reasoning system can reuse already defined ontologies for describing IoT devices and characteristics of persons, adding a link between these two elements in a formal and structured way. The proposed reasoning system can turn an IoT equipped environment into an autonomous and automatic system capable of automatic adaptation of the environment to the personalized concept of comfort of every given user in a holistic way.

### 3.1. General Architecture

**Figure 1:**
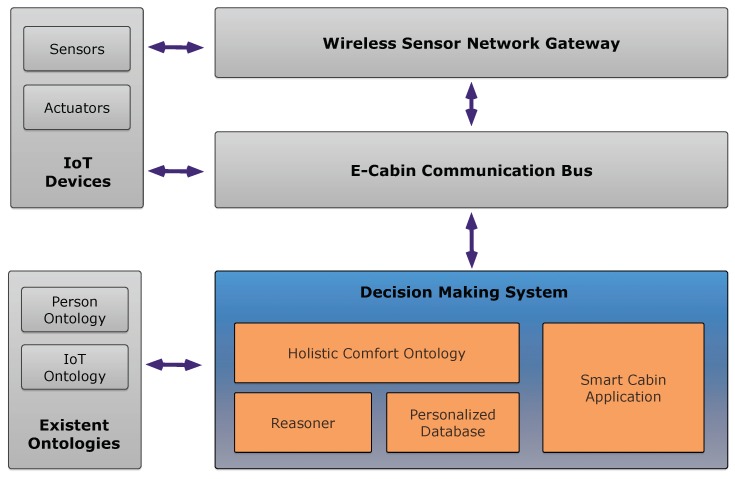
General architecture of the proposed reasoning system.

The system’s architecture leverages is centralized, based on the local network of the cruise ship, and it is managed by the shipowner. More precisely, the shipowner has the knowledge about the status of each cabin of the ship and about the IoT devices installed in each of them. Moreover, as each passenger of the cruise shall enroll to enter the ship, the shipowner can ask them whether they would like to adopt the E-cabin feature; in case of an affirmative answer, the passengers are required to fill in data related on their personal ontology. All data can be stored and managed on a centralized server that operates on the ship network, so all issues related to privacy and security can be addressed as for other sensitive data stored on the central servers. The system is structured as shown in Figure 1.

Figure 1 shows the Decision Making System architecture for a single cruise cabin, which is built on top of existent ontologies (such as person-related and IoT sensors and actuators ontologies). The Decision Making System proposed is connected via the E-Cabin Communication bus to the net of IoT sensors that is equipped inside the cruise cabin.

The data regarding all the E-Cabin equipped cabins and the related passengers data shall be stored in local database inside the ship. More precisely, the personal and sensitive passenger data shall be collected by the shipowner in a private and secure database space that operates on the ship and is managed by the ship owner. The specific characteristics of each IoT device also remain in the shipowner private and secure database space inside the local network of the ship. Thus, all the IoT and persons related data are only accessed locally and they are not transferred to any other party during the reasoning process. For these reasons, the proposed architecture is compatible with the principles of the General Data Protection Regulation (GDPR) that defines personal data privacy management rules in the European Union.

### 3.2. Passenger Ontology

E-Cabin’s passenger is modelled relying on the Friend Of A Friend (FOAF) vocabulary [42], a model that allows for representing passenger’s registry records (with properties like foaf:firstName, foaf:FamilyName, foaf:birthday; foaf:age, etc.). FOAF, developed for describing networks of people, contains all the core elements to describe the “ID card” of a passenger.

**Figure 2:**
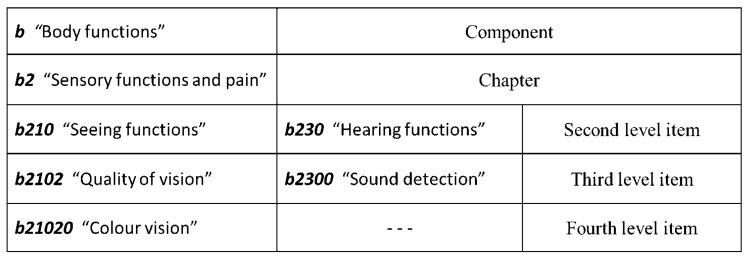
An example of ICF (International Classification of Functioning, Disability and Health) illustrating the structure of the classification.

This domain ontology also assigns each passenger to his/her cabin (i.e., the cabin he/she bought when he/she purchased the cruise); moreover, the passenger can decide whether to specify or not his/her special necessities. This can be done by resorting to the International Classification of Functioning, Disability and Health (ICF) [43], a World Health Organization Standard that allows for describing the functioning of an individual. ICF is divided in four components (Body functions, Body structures, Activities and participation, Environmental factors), each of which is further deepened into Chapters, which identify the addressed domain; each component is identified by a letter (b for Body functions, s for Body structures, e for Environmental factors, d for Activities and participation) and can be deepened by adding digits. According to the number of digits following the letter, it is possible to get a code, whose length indicates the level of granularity—up to five digits (as exemplified in Figure 2).

The functioning or disability of an individual can be assessed selecting the suitable category and its corresponding code and then adding a qualifier (0: no impairment, 1: mild impairment, 2: moderate impairment, 3: severe impairment and 4: complete impairment). ICF has also been represented into a reference ontology [44]: E-Cabin reuses this ontology, limiting the extent of the ICF components to Body functions and Body structure, and allowing for specifying a granularity up to the second level (three digits after the letter of the component).

**Figure 3:**
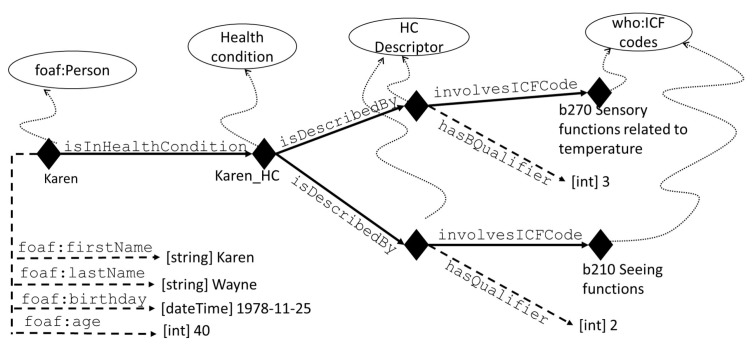
The modelling of a passenger with FOAF and ICF. Individuals are represented with diamonds, full line arrows indicate object-type properties, dashed line arrows indicate datatype properties, curved dotted lines state the type of an individual.

The modelling of the passenger’s domain ontology retraces the ontology design pattern described in Refs. [28,30], where each person’s health condition is described with specific descriptors requiring one ICF code and its qualifier—as depicted in Figure 3:

### 3.3. Sensor and Devices’ Ontology

For modelling sensors and devices inside the cabin environment, E-Cabin relies on BOnSAI [36], an ontology developed for AmI. This model encompasses several subsets of ontologies that allow for describing sensors, actuators and the services the devices provide. Implemented in OWL with Protégé, BOnSAI provides classes to conceptualize context, services, hardware and functionalities by reusing concepts coming from other ontologies—such as Digital Environment Home energy Management System (DEHEMS) [45] and the context ontology CoDAMoS [46].

**Figure 4:**
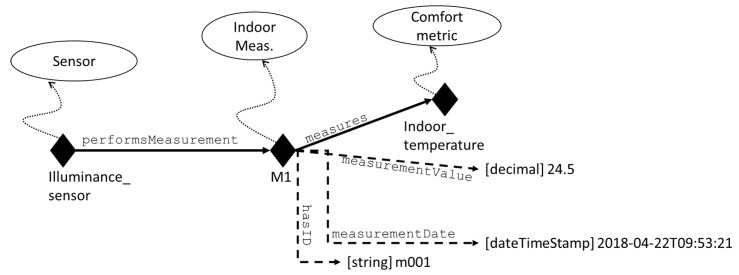
An example of sensor modelling and its measurement.

Considering that E-Cabin does not require modelling household appliances (such as oven, refrigerator, etc.), BOnSAI expressivity is more than enough to cover the cabin’s environment description. Following and ontology design pattern already used in Refs. [28,30], E-Cabin’s ontology adds the possibility to model the measurements performed by the sensors (illuminance sensor, thermo-hygrometric sensor, CO${}_{2}$ concentration sensor) in individuals, each of which is characterized by the value of its measurement and unit of measurement—resorting to a subset of Ref. [47]. Figure 4 provides an example of sensor modelling and its measurement.

### 3.4. Comfort Metrics Ontology

The description of comfort metrics in E-Cabin relies on a domain model developed starting from Ref. [30]. Concepts representing the four comfort metrics monitored inside the cabin environment (CO${}_{2}$ concentration, indoor temperature, indoor illuminance and indoor humidity rate) are modelled, and each of the classes is divided in Comfortable or Uncomfortable. Each measurement performed by the sensors—as described in the previous subsection—is classified according to its value to determine whether there is a comfortable situation or not.

Illuminance comfort is determined modelling the thresholds described in the European norm EN 12464-1, which sets a minimum of 200 lux as a minimum requirement for the occupied environment; for the amount of acceptable CO${}_{2}$ concentration inside the cabin, the ANSI/ASHRAE Standard 62.1-2016 [48] sets the maximum threshold to 1000 ppm. These thresholds can be modified by the passenger in any time in order to determine his/her personal comfort.

Moreover, if the passenger declared that he/she suffers from problems related to respiration (such as those described with ICF code s430 “Structure of respiratory system”) or temperature (b270 “Sensory functions related to temperature and other stimuli”), he/she is asked to specify his/her comfortable range—thus reproducing comfortable conditions inside the cabin. Therefore, each time a measurement exceeds the threshold and becomes uncomfortable, one or more actuators intervene to restore comfortable conditions (for instance, triggering the opening of a window, activating air conditioning, turning lamps on).

### 3.5. Semantic Repository and Reasoning Engine

The E-Cabin ontologies described in the previous subsections are hosted on a triple-store [49], a semantic repository that allows for querying, retrieving and modifying the stored semantic data using the SPARQL Protocol and RDF Query Language (SPARQL) [50]. E-Cabin chose the Stardog repository (version 5.3.4) [51]—a widely used graph platform—since it can support the querying activities and reasoning using the W3C-endorsed languages RDF, OWL and SWRL. Reasoning in Stardog is performed at query-time, which entails that the triples resulting from reasoning (for example, the triples resulting from the application of SWRL) are not materialized, and therefore are not automatically added to the ontology. On the contrary, the reasoner solves only those SWRL rules necessary to solve a query, thus reducing the time dedicated to reasoning processes.

Another interesting feature of triple-stores consists in the possibility to add and modify data using INSERT and DELETE query patterns; in this way, it is possible to customize at any time the comfort metrics personalized by the passengers and the comfort thresholds provided by norms—according to passenger’s desiderata.

E-Cabin application can exchange information with the semantic repository (therefore with the knowledge base) relying on a semantic middleware (described in Ref. [30]), a software developed with Java and run every time the passenger selects an activity to be performed inside the cabin via the smartphone application. Each time a passenger selects an activity, the middleware runs and generates the corresponding SPARQL query to retrieve the (i) passenger’s health condition and (ii) location in the cabin of the passenger. Then, this information is passed from the smartphone app to the middleware using JavaScript Object Notation (JSON) files, thus constituting the input for the SPARQL query. After running the query over the knowledge base hosted on the repository, the latter returns the results (i.e., the comfort metrics to be applied and the actuation necessary to provide the passenger with comfortable conditions while performing the selected activity).

## 4. Cruise Cabin Use Case

The notion of holistic comfort [52]—which follows the idea that the indoor environment has a key role in reaching an adequate level of comfort—arises in the field of nursery care, but can be applied also in other fields where comfort plays an important role. In the context of this work, in which comfort is fundamental, the concept of holistic comfort is applied to the cruise cabin environment—and the research focuses its attention on the cabin areas and the passengers’ comfort. In fact, passenger boarding on a cruise expects a holistic experience that has to satisfy his/her demand and has to offer a wide range of services, products, and experiences. Therefore, cruise ship operators transport passengers by sea for pleasure, and passengers’ comfort is one of their main priorities [53]. The research focuses the attention on the cabin space and its comfort. The cabins available on the market already provide the passengers with a high level of comfort, but their indoor environments cannot be personalized since they are unable to consider passengers’ characteristics and needs. Therefore, by relying on the holistic comfort concept, a higher level of comfort is considered.

Introducing an extended and holistic comfort concept can play an important role as an attraction tool for the more demanding tourists and, consequently, it can be also a plus value for the cruise operators. By reducing the uncomfortable feeling and increasing the comfort perception, the attractiveness of the cruise ship exponentially increases and the Cruise Operators income can consequently increase.

In the scientific research, it is verified that cabin comfort, sizes, amenities and cleanliness are key factors in the accommodation quality evaluation [6]. However, the elements of the cabin (furnishings, materials, sounds, smells, temperature, humidity, ventilation, brightness, hygiene, etc.) are not enough to satisfy the holistic concept of comfort. It is necessary to consider also the passengers’ characteristics, their needs and their feelings in the cabin environment.

To verify this hypothesis, some scenarios and use cases are described. Moreover, the use cases depict the interaction between the passengers and the Smart Cabin. In the following use cases, the cabin is linked to a passenger in order to demonstrate how the smart environment could help the user to perform his/her activities inside the cabin.

The actors of the various scenarios are: the person, the cabin environment, the devices and the DMS. Various attributes are linked to the actors as follows:

1. Person: the Person class has two derived classes (Passengers and Crew) and the following attributes: Age (children, teenager, adult, elderly), Genre (men, woman, other), and Activity (sleeping, reading, light activity);
2. Cabin: the Cabin class has four derived classes (Indoor cabin, outdoor cabin, cabin with balcony and suite) and the following attributes: Position (low deck, high deck, bow, central, stern), Bed (standard, family);
3. Device: the Device class has three derived classes (Sensors, Actuators, and Sensors–Actuators) and the following attributes: Class (environmental, energy consumption, physiological), Quantity (temperature, humidity, luminosity, colour, movement, etc.), and Unity (degrees Celsius, % of Relative Humidity, lux, ecc.)
4. DMS: the DMS class has three derived classes (Automatic, Semi-automatic, and Decision Making Systems).

### 4.1. Scenario 1: Reading Activity in the Cabin Environment

This scenario takes into account the reading activity in the cabin. Different categories of people could be considered, such as young or elderly. Generally, to create a good and efficient reading environment, it is fundamental to delete annoying reflections and eliminate the shaded areas by creating a diffused light in the cabin and a punctual light on the book. Moreover, this scenario has to consider that the necessary light for a comfortable reading varies from person to person; consequently, the light characteristics are a relevant subjective factor.

#### Use Case 1.1: Start of the Reading Activity

The Use Case 1.1 describes the process related to the starting point of the reading activity in the cabin with balcony. The precondition is that the passenger is already registered on the Smart Cabin Application and he/she is logged in (see Section 5 for further details):

1. The person is inside the cabin.
2. The person decides to focus on the reading activity.
3. The person decides the category of reading (study, work, hobby, relaxation) and the type of reading support (book, magazine, newspaper, school text, professional text, digital book).
4. The person chooses the position of reading, for example: sitting at the desk, sitting on the couch, lying on the bed, etc.
5. The person uses the E-Cabin App to set the desired activity: reading. The E-Cabin App combines different sets of lighting parameters related to visual comfort (lighting level, light tone, direction of light, glare, shadow distribution, illuminance distribution) and environmental parameters linked to thermo-hygrometric comfort (temperature, humidity, airflow). The combination of these parameters together with the reading activity and the passenger’s characteristics (visual skills, gender, age, physical impairments, etc.) creates the output for improving the comfort conditions. Moreover, in the case of background noise, or if the person prefers to read with music or with background music, the E-Cabin App sets a suitable sound environment.
6. The passenger moves from the “non-reading” phase to the “start reading” phase.

**Figure 5:**
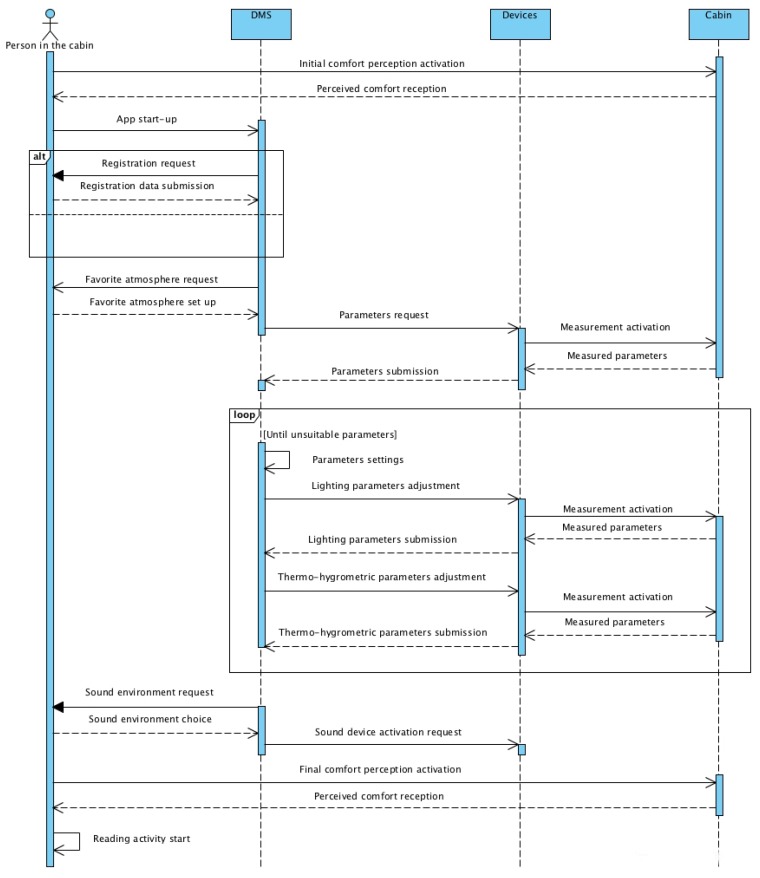
Sequence diagram for the Use Case 1.1.

Figure 5 shows the sequence diagram of the Use case 1.1. In the unified modeling language (UML) diagram, four specific objects (person in the cabin, DMS, devices and cabin) are reported with the respective connections. The passenger perceives a variation of comfort due to the sequence of adjustments imposed by the DMS to the devices to regulate comfort.

### 4.2. Scenario 2: Sleeping Activity in the Cabin Environment

This scenario takes into account the adaptation of comfort conditions for the passenger who wants to perform a sleeping activity in the cabin. The cabin settings adapts automatically to the person’s needs; in particular, the activity linked to awakening from sleep at a set time, may involve different categories of people, for example young or elderly. Consequently, also the awakening process has to be personalized as follows:

- Acoustic alarm (defined by a sound signal, music or radio station);
- Luminous alarm (defined by changing the intensity and colours of different lights);
- Combination of acoustic and luminous alarms.

#### Use Case 2.1: Forced Awakening Due to a Fixed Commitment (Such as an Excursion)

The Use Case 2.1 describes the process related to the passenger’s activity who has to wake up at a certain time, for example, for an excursion organized by the cruise company. Even in this Use Case, the precondition is that the person is already registered on the Smart Cabin Application, he/she is logged in, and he/she has already set the alarm on the Smart Cruise App (see Section 5 for further details):

1. The Smart Cabin Application recognizes the person in bed.
2. The person wakes up autonomously: the Smart Cabin Application detects it and cancels the awakening procedure.
3. The person is in the bed at the predefined time: the Smart Cabin Application activates the awakening procedure.
4. The application creates the personalized comfort environment and by doing so facilitates the user’s awakening. Lighting comfort, thermo-hygrometric comfort and acoustic comfort are combined with the activity and the user’s characteristics (visual skills, gender, age, physical impairment, etc.) for optimizing the awakening process.
5. The person delays the alarm: the Smart Cabin Application sets a new procedure for waking up.
6. The person gets up: the Smart Cabin Application detects the movement and ends the awakening procedure.

**Figure 6:**
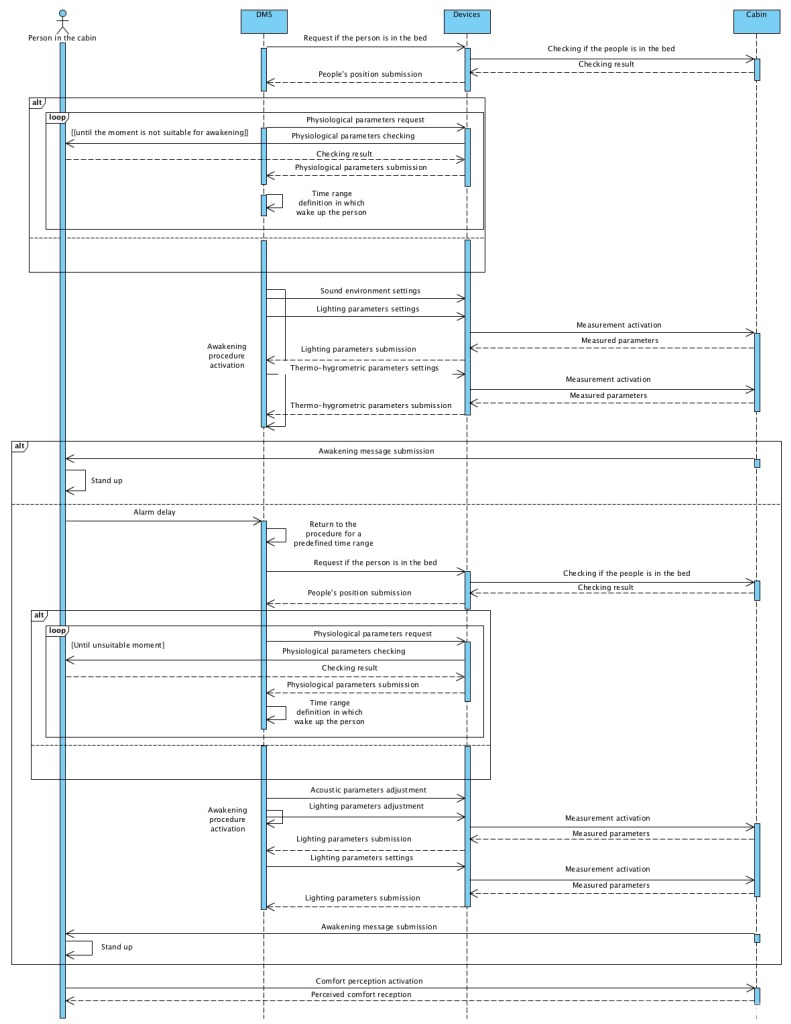
Sequence diagram for the Use Case 2.1.

Figure 6 shows the sequence diagram of the Use Case 2.1. In the UML diagram, four specific objects (person in the cabin, DMS, devices, and cabin) are reported with the respective connections. The passenger perceives a variation of comfort due to the sequence of adjustments imposed by the DMS to the devices to regulate comfort.

### 4.3. Scenario 3: The Environment Changes Due to the Passenger’s Absence

**Figure 7:**
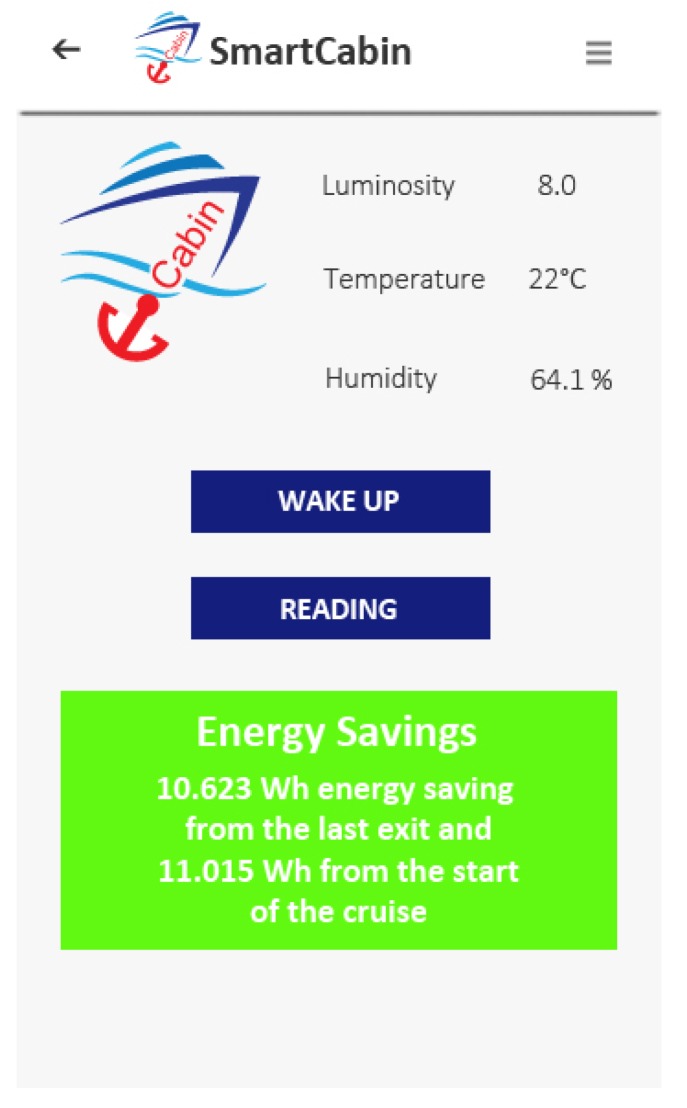
Energy savings message.

This scenario is focused on the energy efficiency and takes into account the passenger’s activity of entering or exiting his/her cabin. During the passenger’s absence from the cabin, a new scenario aimed at saving energy is set. The person has to wear the bracelet/beacon during the period of absence from the cabin. In this way, the passenger’s position can be detected and the system can both set the cabin in the energy saving scenario—in case the person exits from the cabin—and restore the state of the cabin before he/she enters. Ancona et al. [54] pointed out the importance of maximizing the energy efficiency and minimizing the thermal energy dissipation. In this research, to make the passenger more sensitive about this topics, a pop up message appears on the smartphone or tablet when the person re-enters in the cabin (Figure 7).

The message provides the passenger with the partial energy savings (accumulated in the time interval of passenger’s absence from the cabin) and the total energy savings (calculated from the beginning of the cruise). Moreover, the data describing the energy savings are read through the middleware and they can be made available also to the Cruise Company.

#### 4.3.1. Use Case 3.1: Energy Saving after a Passenger Leaves His/Her Cabin

The Use Case 3.1 describes the process concerning the passenger’s temporary absence from the cabin and the consequent reorganization of the energy saving scenario. The precondition for this use case is that the person is already registered on the Smart Cabin Application, he/she is logged in, and he/she wears the bracelets/beacon:

1. The person leaves the cabin.
2. When the person reaches a certain distance from the cabin, the DMS starts the procedure for energy saving scenario. By combining different sets of lighting parameters and environmental parameters, the cabin environment is reconfigured with metrics aimed at saving energy.
3. The Cabin is in the Energy Saving modality.

#### 4.3.2. Use Case 3.2: Cabin’s Indoor Comfort Restoration Just before the Passenger’s Return

The Use Case 3.2 describes the process concerning the cabin restoring tailored indoor comfort metrics due to passenger’s return to the cabin. The precondition is that the person is already registered on the Smart Cabin Application (see Section 5 for details), he/she is logged in, he/she wears the bracelets/beacon, and he/she has left the cabin before and he/she is returning to his/her cabin:

1. The person decides to return to the cabin.
2. The person is identified (by smart devices installed in the public spaces) near his cabin.
3. The DMS reactivates the cabin’s settings that were in force before the passenger left the cabin.
4. The person enters in the cabin and finds the same environmental configuration that he/she had left before exiting.

**Figure 8:**
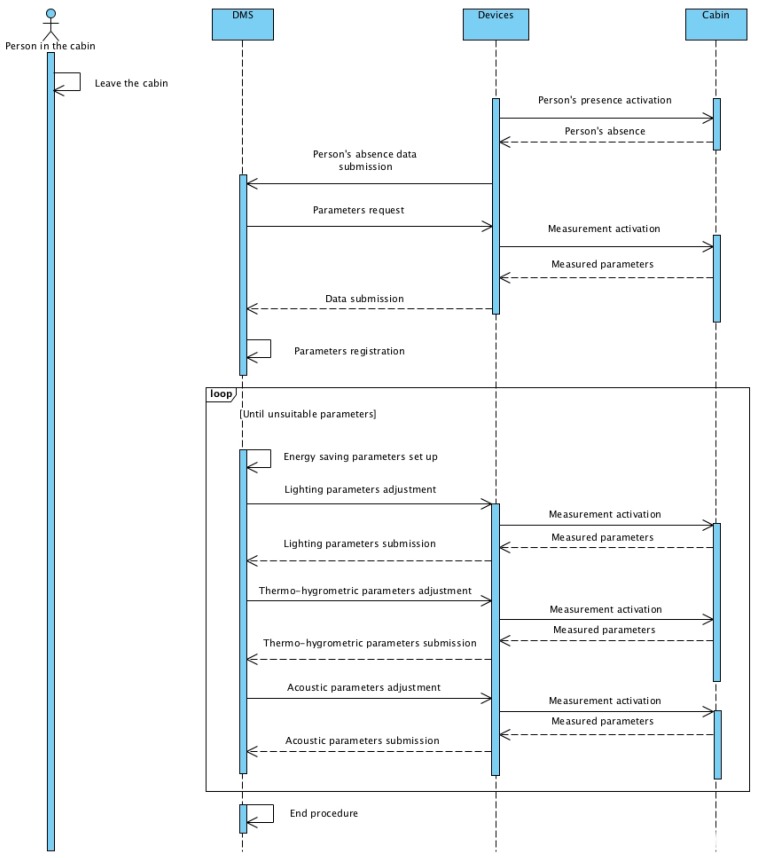
Sequence diagram for the Use Case 3.1.

**Figure 9:**
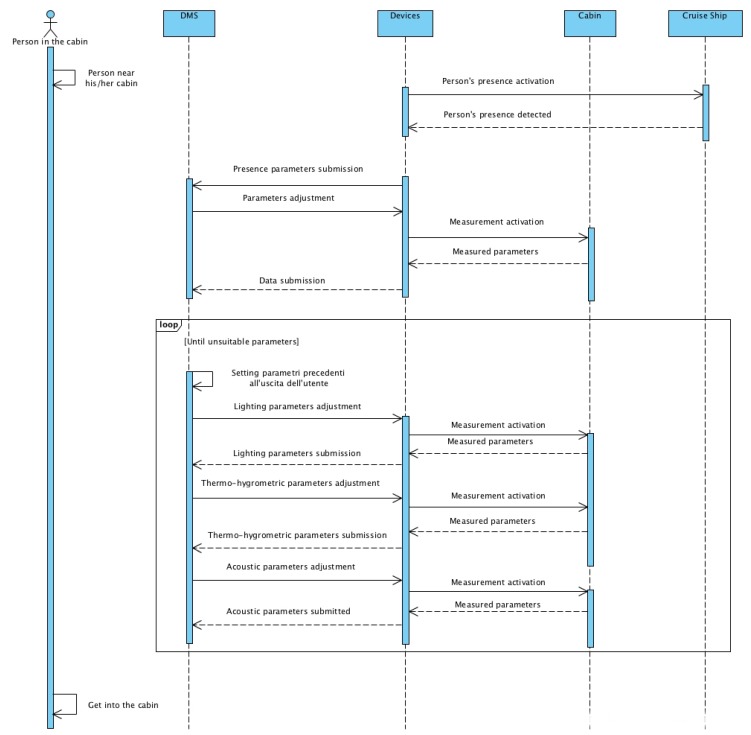
Sequence diagram for the Use Case 3.2.

Figure 8 and Figure 9 show the sequence diagram of the Use Cases 3.1 and 3.2. In the UML diagram, four specific objects (person in the cabin, DMS, devices, and cabin) are reported with the respective connections. The cabin perceives a variation of status due to the sequence of adjustments imposed by the DMS to the devices to adapt to the new scenario.

## 5. Smart Cabin Application

Smart Cabin Application has been designed to monitor and regulate the environmental parameters and, consequently, to guarantee to the passengers the best performance of comfort (visual, acoustic and thermo-hygrometric) during various activities (for example, sleeping, reading and changing clothes) carried out in the cruise cabin. Moreover, the application presents an added value compared to the domotic systems currently on the market because it proposes an integrated system—the E-Cabin platform—that allows the integration of heterogeneous sensors and actuators in a unified way. It implements a personalized comfort in the cabin based on the characteristics of the passenger through queries to the reasoner and the use of basic knowledge. After the Application’s download from the store, it is necessary to launch the application and start the registration phase.

As a first step, the cabin number is requested. In this way, the system is able to find all the information that the passenger stated during the check-in; all these data are comprised in the semantic repository. As a second step, the passenger can provide additional information on a web page that structures the personalized database. The stated data are reported in Table 1.

### 5.1. Smart Cabin Architecture

As introduced in the previous section, the Smart Cabin App is an application that works as a Decision-Making System. The user can monitor the status of the cabin by using the GUI (Graphical User Interface) of the App. The App is connected to the semantic repository and all the smart devices installed in the cabin environment through the E-Cabin platform. The App architecture involves four elements that interact among each other. More precisely, the elements in the App architecture are:

- DMS module;
- Data Base;
- Graphical User Interface (GUI);
- Interface with the E-Cabin platform.

**Figure 10:**
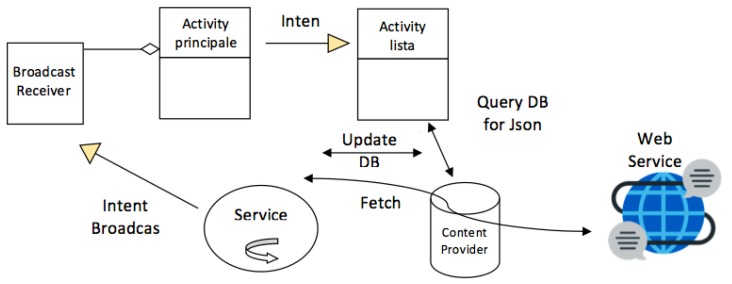
Smart cabin architecture.

The E-Cabin platform is based on a publisher–subscriber communication framework. The E-Cabin platform provides Topics and Messages for interfacing with IoT sensors and actuators, and with the reasoner. The structure and logic of the Smart Cabin App architecture are described in Figure 10. The Service components and the Content Provider are constituted by an independent module. The Service tasks are to notify the main activity on the presence of a Bluetooth Low Energy (BLE) beacon. The notification system is based on Intent, and, in the receiving Activity, provides a Broadcast Receiver for the presence. The Service has also another task; based on the information provided by the external Web Service, the Service updates the data in the application database. The database is managed through the Content Provider. In this way, the access and the use of the various components of the application are safe.

### 5.2. Smart Cabin Application User Interface

The layout, aesthetically pleasing and appealing, and the graphic interface, intuitive and functional, are designed to improve the visual comfort of the end user. This section describes the main features of the application divided in: log in, default settings, home page, and main menu.

#### 5.2.1. Log in

**Figure 11:**
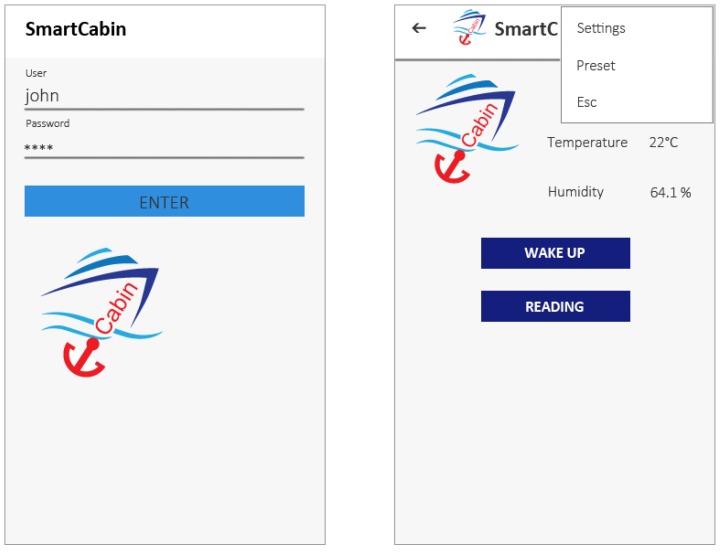
Log in screen-shoot (on the **left**) and Home Page screen-shoot (on the **right**) of the Smart Cabin Application.

When the application is launched, a login screenshot appears on the screen (Figure 11). The user, already registered on the E-Cabin system, can insert the nickname and the password.

#### 5.2.2. Default Cabin’s Settings

After the login procedure, the default settings of the cabin are activated. The indoor atmosphere deployed is personalized based on the user’s characteristics that have has just been logged in.

#### 5.2.3. Home Page

After the log in procedure, the main home page appears as shown in Figure 11. The GUI has grid’s disposition of elements, which facilitates the users in the accessing, understanding, and using the application [55]. The Home page layout is divided into three main parts: weather station, menu and activities.

**Figure 12:**
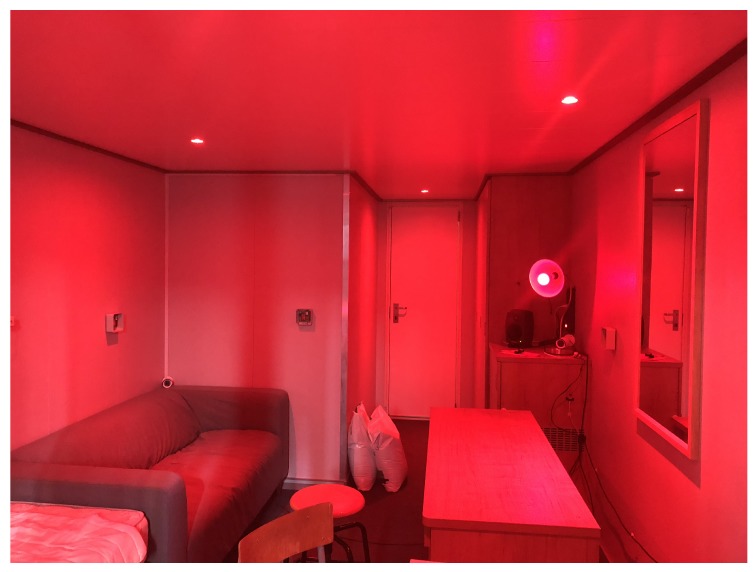
Demo Cabin: red light atmosphere.

In the following paragraph, the main parts composing the home page structure are described:

- Weather Station: The temperature, humidity, and illuminance are reported in real time. Since the cabins are equipped with various ambient sensors that monitor the environmental status, it is possible to provide this information to the passenger.
- Activities: This section allows the passenger to choose the desired activity (e.g., reading on the sofa, reading in bed and waking up). This section, with the appropriate cabin equipment, could disappear as a consequence of a full automated cabin.
- Menu: The Menu button links to another screen with additional features (Settings, Preset and Exit). In the following paragraph, the functionalities of the buttons are described.
  1. Exit: this button closes the application;
  2. Settings: this section allows the user to modify some settings. The user can turn on/off the lights, log out or set the initial cabin “preset” with the default cabin’s settings (already described in Section 5.2.2).
  3. Preset: this section allows the user to customize the atmosphere in the cabin. He/She can change some parameters of the various actuators by using the application on the smartphone or tablet. For example, as shown in Figure 12, the passenger can change the colour and intensity of the light and can decide to turn the music on/off.

## 6. Test and Validation

The development of the above-mentioned application constitutes a plus value compared to the domotic systems available on the market, since it enables the adaptation of the comfort conditions according to a guest’s current activity and to his/her preference. To verify this hypothesis, a double typology of tests has been performed. The application test has been used to validate the use cases, while the subjective test has been done to understand if users find it useful to have a cabin where the environment changes according to their activities and characteristics.

### 6.1. Subjective Test

The subjective test presented in this research is based on the participants’ opinion and involves their personal feelings about comfort in the cruise cabin. The test has been done to understand if users are familiar with smart devices and if they consider it useful that the environment changes according to their characteristics and their different activities in the cabin.

#### 6.1.1. Participants

Thirty adults—13 males and 17 females—were enrolled in the study. The people interviewed are representative of the Italian working class, with a medium-high level of education, an average age of about 45 (14 is under 40, 16 are over 40). Everyone declared a good propensity to travel. Except for two persons, the interviewees did not attend cruises. They all were in a good general health status. Exclusion criteria were: moderate to severe motor impairment or vision impairment; cognitive decline; inability to read Italian language; and inability to provide informed written consent. Table 2 reports all other demographic characteristics of the study participants. The subjects participating in the study provided informed written consent.

#### 6.1.2. Equipment

**Figure 13:**
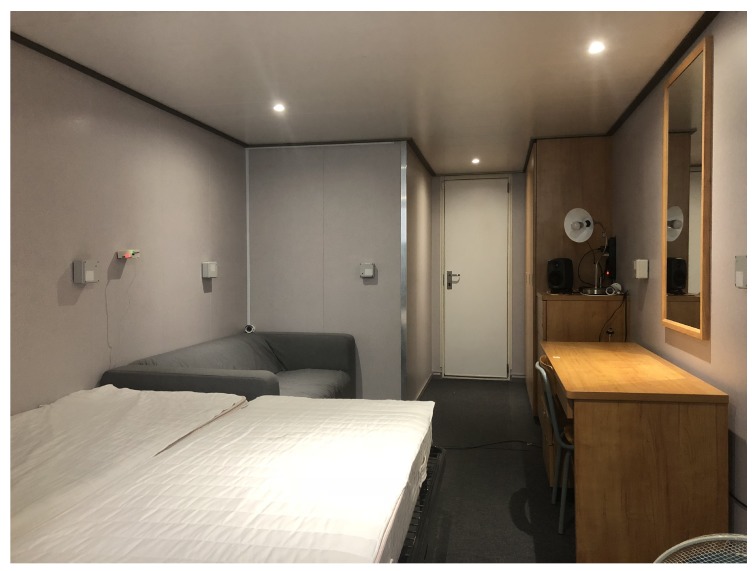
Cabin environment. The Demo Cabin was provided by Fincantieri S.p.A.

The test took place in a Demo Cabin (Figure 13). The cabin is divided into four functional areas: sleeping area, living area, bathroom (not accessible area) and entrance. The guest can enter in the cabin only through the balcony door, as the entrance door is not accessible. Consequently, the guest is immediately in the sleeping area and then continues to the living area, where the test took place. The cabin dimensions—including the bathroom—were 3 × 6 m${}^{2}$, and cabin height was 2.20 m.

The cabin’s four functional areas were furnished as follows:

- Entrance: wardrobe, minibar;
- Sleeping area: bed 1, bedside table 1;
- Living area: chair 1, desk 1, minibar 1.

The division of the spaces and the furniture organization correspond to the characteristics of the environment described in the ontology. The cabin was also equipped with networked devices able to monitor the environment and to provide adequate comfort metrics according to each passenger’s preferences and to the activity he/she is performing.

#### 6.1.3. Protocol

Each subject performs the test in the above-mentioned cabin environment. Each session started when the user was seated on the chair facing the desk. The user received the smartphone with the Smart Cabin Application. The user autonomously discovered the functions of the application. The test procedure ended with the questionnaire.

#### 6.1.4. Measures

To evaluate the passenger’s comfort, an ad hoc questionnaire was developed. The questionnaire provides two items aimed at investigating the knowledge and the use of smart devices and two items focused on the usefulness of self-regulation.

The questionnaire consisted of the following questions:

**Q1**  How much do you use IoT devices (e.g., Smart Home devices) in your daily life?
**Q2**  Do you feel comfortable with the smart devices in the cabin?
**Q3**  Do you find useful that environmental conditions adapt to the activity that you do?
**Q4**  Do you find pleasing the auto-regulation of the environment regarding your activities?

For all questions, the user had to indicate his/her level of agreement on a 7-point Likert scale ranging from 1 (strongly disagree) to 7 (strongly agree).

#### 6.1.5. Statistical Analyses and Results

**Figure 14:**
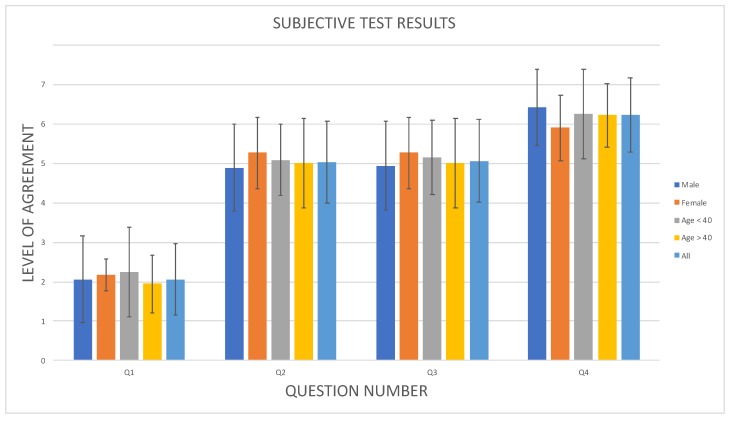
Subjective test results. The values reported in Table 3 are depicted in graphical format. The solid bars represent the mean value and the lines the standard deviation of the level of agreement (from 1 to 7) for the reply of the following five categories: male, female, age below 40, age above 40, all the persons in the sample.

Data resulting from the questionnaire were analyzed using one-way repeated measures ANOVA. Results of the questionnaires for the considered activity are reported in Table 3 and are graphically depicted in Figure 14. The results are presented taking into account the following categories: male, female, age below 40, age above 40, and all the sample (all in Table 3).

### 6.2. Application Test

The application test presented in this research is based on the functionality and activities of the Smart Cabin Application described in Section 5. The aim of the Smart Cabin Application is to monitor, regulate and guarantee the best comfort performance (visual, acoustic and thermo-hygrometric) to the passenger during various activities carried out in the cruise cabin. The test has been done for testing the Use Case activities described in Section 4.

#### 6.2.1. Participants

Three adult profiles—two males and one female—between 25 and 46 years old have been considered in the study. They all were in good general health status. Exclusion criteria were the same of the criteria described in Section 6.1.1.

#### 6.2.2. Equipment

The test took place in the same Demo Cabin described in the Section 6.1.2 of the Subjective test.

#### 6.2.3. Protocol

Each subject performs the tests in the above-mentioned cabin environment. The user received the smartphone with the Smart Cabin Application. Each session started after the user’s log-in (the registration phase had already done). The user independently discovered the activities of the application and simulated the four Use Cases. The test procedure ended with the evaluation of the Use Cases, e.g., if the Use Case 1.1 was completed or not.

#### 6.2.4. Measures

To evaluate the Use Case activities of the Smart Cabin Application, all three of the user performed the following Use Cases. Every user repeated the procedure a fixed number of times. For every repetition, the user had to indicate the success/failure of the process—for instance, if the environment changed considered his/her activity and characteristics.

#### 6.2.5. Results

A total of 139 executions were carried out on Smart Cabin Application for testing the Use Case activities described in Section 4. All executions were successfully completed as shown in Table 4.

### 6.3. Discussion of Test Results

The results of the two tests show that the tested users seldom use IoT smart devices different from the smartphone in their daily life. Although the IoT devices are rarely used, these users have a strong interest in spending time in cabins equipped with IoT devices. In the tested sample, there is no noticeable difference in the response between males and females, although men are a little more interested in using personalized comfort adjustment. Note that, although older people are slightly less accustomed to using IoT devices, they also have a considerable interest in customizing cabin comfort.

The three different Use Cases and the three different user profiles helped us in testing the Smart Cabin Application in different operational conditions. For each of the user profiles, the test was repeated several times, to check if the system response is repeatable. The Smart Cabin Application successfully fulfilled the tested Use Cases, showing that three different user profiles can be managed, and that personalized comfort configurations are inferred and then applied nearly in real time.

## 7. Conclusions and Future Works

In this paper, we presented a novel decision-making system capable of optimizing the cruise cabin comfort. This system leverages an ontology-based representation of the passengers and their health condition, indoor comfort metrics and sensors and actuators to provide tailored comfort adaptations to the passengers, exploiting reasoning techniques enabled by Semantic Web. The main novelty of the decision-making system relies on the definition of a new comfort metric domain ontology, mainly based on the concept of holistic comfort, which is able to reuse already defined and well-known person and IoT ontologies. In fact, exploiting semantic reasoning, the system can automatically adjust the comfort for a passenger, knowing his/her characteristics and taking into account the activity the person is performing in a particular area of the cruise cabin. A mobile-device application acts as an interface to allow the passenger to communicate with the decision-making system, so that he/she can select the activity he/she wants to perform, while hiding the complexity of the actuation performed in the cabin environment. Subjective results show a high acceptance rate of the automatic comfort optimization based on holistic comfort, while repeated tests show that the implemented demo scenarios are implemented in a reliable and affordable way.

Future works will consider a wider set of activities, extending the range of the use cases considered. New use cases will be drafted to consider other different IoT equipment and devices inside the cruise cabin. Among these activities, the possibility to provide personalized music to the passenger can also be evaluated; this would require a modification of the ontological framework to also encompass passengers’ musical preferences. Another research line for the proposed framework is to investigate whether it could be adopted in different environments of the cruise ship, such as private areas inside restaurants, SPA areas, bars, etc. Moreover, the proposed decision-making system could be compared to the "Comfort as a Service" (CaaS) paradigm [56], which releases indoor environments’ occupants from managing and operating with the comfort equipment with the aim of reducing energy wasting, increasing costs and uncomfortable indoor metrics. In relation to the presented framework, the IoT devices can enable the idea of CaaS.

Finally, in order to assess the passengers’ acceptance for these kinds of technologies, the standard Technologies Acceptance Model (TAM) [57] and System Usability Scale (SUS) [58] can be adopted as tools to evaluate the system.

## Figures and Tables

**Table 1:** Passenger’s characteristics.

Semantic Repository	Personalized Data Base
Name and surname	Favorite colour
Gender	Preferred atmosphere
Date of birth	Favorite music genre
Disability (motor, auditory, visual etc.)	Smoker
Special needs (for example if he is vegetarian)	Sport

**Table 2:** Sample’s characteristics.

Parameters	Value
Participants (number)	30
Gender (male/female)	13/17
Age	44 ± 18
Education (years)	15.6 ± 3

**Table 3:** Subjective test results. The mean value and the standard deviation of the level of agreement, ranging from 1 (strongly disagree) to 7 (strongly agree), has been computed for each question and for each category.

Question	Mean Value					Standard Deviation				
	Male	Female	Age < 40	Age > 40	ALL	Male	Female	Age < 40	Age > 40	ALL
Q1	2.00	2.18	2.25	1.94	2.04	1.10	0.40	1.13	0.72	1.50
Q2	4.89	5.27	5.08	5.00	5.04	1.10	0.90	0.90	1.13	1.62
Q3	4.94	5.27	5.17	5	5.04	1.13	0.90	0.94	1.14	1.62
Q4	6.41	5.91	6.25	6.22	6.29	0.96	0.83	1.14	0.81	1.24

**Table 4:** Application test results. We have considered six different test cases, and in each of them we have tested the Application using three different user profiles, called Profile1, Profile2 and Profile3 in the table. Each user profile corresponds to a different configuration in the knowledge base. For each profile and each use case, two numbers are reported: the number of successful trials and the total number of trials for that test. The overall number of successful trials and the number of total trials for a given Use Case is finally reported in the Results column.

Use Case	Test			Results
	Profile1	Profile2	Profile3	
1.1.1—Start of the reading activity on the sofa	10/10	9/9	8/8	27/27
1.1.2—Start of the reading activity on the bed	8/8	10/10	8/8	26/26
2.1—Forced awakening due to a fixed commitment (ex: excursion)	10/10	10/10	10/10	30/30
3.1—Energy saving cabin settings after the exit of the passenger	10/10	9/9	9/9	28/28
3.2—Cabin environment restoration after the energy saving	10/10	9/9	9/9	28/28
scenario and just before the passenger’s entrance				

## Abbreviations

The following abbreviations are used in this manuscript:

AAL	Ambient Assisted Living
Ami	Ambient Intelligence
App	Application
BLE	Bluetooth Low Energy
CA	Context Awareness
DEHEMS	Digital Environment Home energy Management System
DMS	Decision Making System
ICF	International Classification of Functioning, Disability and Health
IoT	Internet of Things
JSON	JavaScript Object Notation
OWL	Ontology Web Language
RDF	Resource Description Framework
SSN	Semantic Sensor Network
SWRL	Semantic Web Rule Language
UML	Unified Modeling Language
